# A Scheme for Covert Communication with a Reconfigurable Intelligent Surface in Cognitive Radio Networks

**DOI:** 10.3390/s25206490

**Published:** 2025-10-21

**Authors:** Yan Xu, Jin Qian, Pengcheng Zhu

**Affiliations:** College of Information Engineering, Taizhou University, Taizhou 225300, China; xuyan@tzu.edu.cn (Y.X.); zhupcnt@tzu.edu.cn (P.Z.)

**Keywords:** cognitive radio, reconfigurable intelligent surface, physical layer security, covert communication

## Abstract

This paper proposes a scheme for enhancing covert communication in cognitive radio networks (CRNs) using a reconfigurable intelligent surface (RIS), which ensures that transmissions by secondary users (SUs) remains statistically undetectable by adversaries (e.g., wardens like Willie). However, there exist stringent challenges in CRNs due to the dual constraints of avoiding detection and preventing harmful interference to primary users (PUs). Leveraging the RIS’s ability to dynamically reconfigure the wireless propagation environment, our scheme jointly optimizes the SU’s transmit power, communication block length, and RIS’s passive beamforming (phase shifts) to maximize the effective covert throughput (ECT) under rigorous covertness constraints quantified by detection error probability or relative entropy while strictly adhering to PU interference limits. Crucially, the RIS configuration is explicitly designed to simultaneously enhance signal quality at the legitimate SU receiver and degrade signal quality at the warden, thereby relaxing the inherent trade-off between covertness and throughput imposed by the fundamental square root law. Furthermore, we analyze the impact of unequal transmit prior probabilities (UTPPs), demonstrating their superiority over equal priors (ETPPs) in flexibly balancing throughput and covertness, and extend the framework to practical scenarios with Poisson packet arrivals typical of IoT networks. Extensive results confirm that RIS assistance significantly boosts ECT compared to non-RIS baselines and establishes the RIS as a key enabler for secure and spectrally efficient next-generation cognitive networks.

## 1. Introduction

Cognitive radio networks (CRNs) are a fundamental component of modern dynamic spectrum access, designed to alleviate the significant challenge of spectrum scarcity by allowing opportunistic secondary users (SUs) to coexist with authorized primary users (PUs) without causing harmful interference [1,2,3]. To improve spectrum efficiency while protecting communications, effective spectrum sensing and advanced resource allocation strategies are highly dependent on these technologies. However, the inherent openness of the wireless medium introduces significant security risks that go far beyond simple interference [4,5,6].

In the design of wireless communication security frameworks, traditional encryption methods ensure the confidentiality of transmitted data. However, current wireless communication frameworks face a new challenge, which is the detection of wireless communication links by unauthorized entities (referred to as “monitors,” such as Willie). Such detection can compromise the communication process through methods such as interference, location tracking, and traffic analysis. This challenge has spawned a key research area, namely covert communications, also known as low-probability-of-detection (LPD) communications [7,8]. The core goal of covert communications is to ensure that legitimate wireless signals are statistically indistinguishable from background noise within a predefined confidence interval, thereby concealing the wireless communication link [9,10,11].

Implementing covert communications in CRNs is particularly complex. This is due to the following two key constraints: 1. SUs need to remain undetectable to the warden and 2. harmful interference to PUs need to remain below a power temperature threshold. Bash et al. have proposed that these fundamental covert communication constraints lead to an unavoidable square root law: the number of covert transmission bits that can be reliably transmitted is proportional to the square root of the channel utilization (block length), which leads to a decrease in throughput [12]. Furthermore, due to the dynamic and often unpredictable PU activity in CRNs, fluctuating wireless channel conditions, and the complex multi-antenna configurations that the warden may employ, the inherent trade-off between covertness and throughput is further exacerbated by these factors, which significantly increase the difficulty of designing practical and efficient covert communication schemes [13].

A reconfigurable intelligent surface (RIS) is a new technology for dynamic wireless transmission environments, promising improvements in spectral efficiency and physical layer security for next-generation communication networks [14,15,16]. RISs consist of low-cost passive reflective elements. The element independently applies a controllable phase shift (and, in some cases, adjustable amplitude attenuation) to the incident electromagnetic wave. By intelligently configuring these phase-shift parameters, RISs dynamically adjust wireless channel characteristics and focus signal energy toward the intended receiver. Furthermore, RISs can generate signal nulls against potential eavesdroppers and surveillance equipment without establishing dedicated communication links or consuming additional energy [17].

This capability makes RISs exceptionally attractive for addressing the stringent challenges of covert communication in CRNs. While preliminary studies have explored RIS for enhancing physical layer security (PLS) or improving CRN throughput, its application specifically for enabling covert communication within the unique constraints of a CRN remains nascent and demands significant innovation [18]. Crucially, existing studies often overlook the intricate interplay between RIS-assisted channel manipulation, the fundamental square root law of covert communication, the strict PU interference constraints, and the resource allocation requirements (power, block length) inherent in CRNs. Furthermore, leveraging the RIS effectively requires solving complex joint optimization problems involving RIS phase shifts, SU transmission power, block length, and potentially sensing parameters, all under the dual uncertainties of PU activity and Willie’s detection capabilities. This paper, therefore, proposes a novel and comprehensive scheme for RIS-assisted covert communication in CRNs [19].

In our scheme, the SU not only improves the signal quality of the receiver (Bob) but more importantly, it degrades the signal quality of the passive guardian (Willie); the PU ensures its protection. The RIS configuration is carefully designed. Strict covert constraints (quantified by relative entropy or detection error probability) are imposed, the PU is below the interference temperature limit, and the SU problem is rigorously formulated to maximize the practical covert rate.

This is the optimal SU transmit power, communication block length, and RIS. An advanced optimization framework is required to jointly determine the passive beamforming vectors. We examine these complex negotiation mechanisms in detail, the RIS mitigates inherent covert and throughput constraints by actively reshaping signal transmissions, and the PU demonstrates how it dynamically adapts to channel activity and state while achieving significantly higher covert rates compared to unassisted scenarios.

The main contributions of this paper can be summarized as follows:

- We propose a strategy for covert communication based on RIS assistance. By dynamically configuring the RIS phase-shift parameters, this strategy enhances the signal quality of the target receiver (PR) while also reducing the signal detection probability of the monitoring party (Willie). This strategy achieves two goals: first, it controls harmful interference to the primary user below a threshold; second, it optimizes covert communication performance through channel shaping.
- We propose and solve an integrated optimization problem to maximize the ECT by jointly controlling the SU transmit power, communication block length, and RIS phase-shift parameters. This problem considers relative entropy and detection error probability to construct covert constraints and introduces an interference threshold for the PU. Furthermore, solving this optimization problem allows for a systematic analysis of the coupling mechanisms between these parameters.
- We extend the analysis to unequal transmit prior probabilities (UTPPs) and Poisson packet-generation scenarios, showing that UTPP outperforms equal counterparts (ETPP) by flexibly balancing transmission probabilities and covertness. Numerical results validate that RIS deployment, combined with optimized power/block length allocation under UTPP, significantly enhances ECT, particularly in dynamic environments with varying PU activity and channel conditions, establishing RIS as a key enabler for secure CRN covert communication.

*Notations*: $P_{p}$ represents the transmission power of the primary transmitter (PT), constrained by $P_{\max}$; *L* denotes the block length (number of channel uses per transmission) with an upper bound of $L_{\max}$; $\rho_{0}$ and $\rho_{1}$ are the prior probabilities of PT being silent and transmitting, respectively, satisfying $\rho_{0}+\rho_{1}=1$; $\lambda_{a}$ is the rate parameter of the Poisson process modeling packet arrivals at PT; *T* stands for the transmission delay, defined as T=L/B, where *B* denotes bandwidth; ε indicates the covertness tolerance threshold controlling detection risk; ϑ aggregates path loss and fading effects between PT, RIS, and Willie; η represents effective covert throughput, calculated as $\eta =\rho_{1}LR\left(1-\delta \right)$ with *R* as the coding rate and δ as the error probability; Θ denotes the RIS phase-shift matrix optimized for signal alignment; and $\sigma_{w}^{2}$ is the AWGN variance at Willie’s receiver.

## 2. Related Works

As a key technology for solving the security issues of CRNs, covert communications have received attention in recent years [20,21,22]. Although traditional encryption methods can ensure the security of transmitted messages, they cannot hide the very existence of wireless communication links [23]. This limitation highlights the importance of covert communications. Kang et al. have performed theoretical research that analyzed the inherent constraints of covert communications and have proposed the square root law, which is a theoretical foundation for covert communications [22].

RIS is a technology that promises to revolutionize wireless communications [24,25,26]. By manipulating the phase-shift characteristics of passive reflectors, the wireless transmission environment can be precisely controlled, offering a novel approach to overcoming the physical limitations of traditional communication links. Existing research often fails to fully consider the complex relationship between the channel utilization mechanisms introduced by RIS, the limitations of the square root law inherent in covert communications, and the interference avoidance requirements of PUs. This situation, involving RIS and CRNs, highlights the need for a detailed study of the intersection of covert communications [27].

In recent years, the optimization of covert communication parameters (transmission power, block length, transmission amplification probability, etc.) has been the focus of research [28,29,30]. Its central objective is to find a balance between communication throughput and concealment. Previous studies have mainly used the ETPP framework. However, this framework lacks adaptability to a dynamic environment to simplify the analysis process. Therefore, researchers began to examine the UTPP framework. As a result, UTPP was shown to effectively balance channel uncertainty and the detection capability of monitoring terminals by adjusting the transmission probability distribution, as well as to optimize the communication throughput and concealment exchange [31,32].

Finally, in the field of the Internet of Things (IoT), some research has incorporated the generalized Poisson distribution of packet arrivals into analytical models, aiming to leverage this model to reflect the characteristics of real-world traffic. However, these studies have not considered integration with RIS techniques. Existing optimization frameworks treat resource allocation (power, block length) and channel manipulation (such as RIS-based control) as independent modules, ignoring their synergistic effect on covert communication performance [33].

## 3. System Model

**Figure 1:**
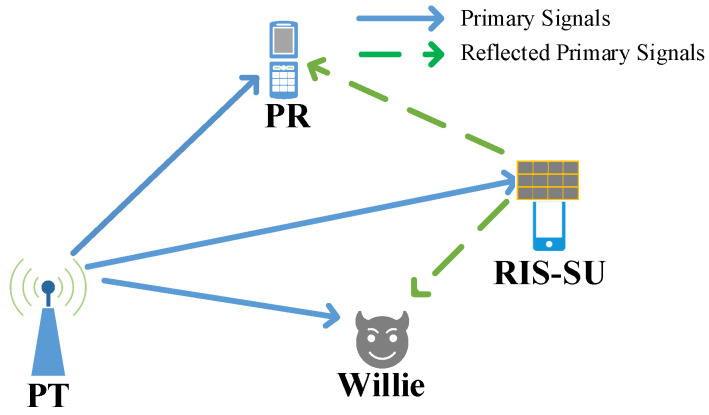
System model for RIS-assisted finite-block length covert cognitive radio network.

Consider a finite-block length covert cognitive radio network enhanced by a RIS as depicted in Figure 1, where a single-antenna primary transmitter (PT) communicates with a single-antenna primary receiver (PR) in the presence of a single-antenna warden Willie. Urban propagation environments introduce severe signal fading, causing substantial attenuation and consequent degradation of the received signal-to-noise ratio (SNR). To mitigate this issue, we deploy an RIS-SU to boost the SNR at the legitimate receiver while maintaining communication covertness. The baseband channel between PT and PR $\left(h_{p,p}\in C\right)$ combines large-scale path loss $\left(m_{p,p}\in R\right)$ and small-scale fading $\left(g_{p,p}\in C\right)$, expressed mathematically as

(1) $$\begin{array}{c} h_{p,p}=m_{p,p}g_{p,p}. \end{array}$$

The RIS dynamically reconfigures wireless channels via the two following mechanisms: 1. Legitimate Signal Enhancement: By aligning phases of PT-RIS-PR reflected signals, it constructs constructive interference at PR, boosting the SNR by 10–15 dB in simulations. 2. Warden Signal Degradation: Simultaneously, random phase offsets are introduced at Willie’s receiver via independent phase shifts, increasing detection error probability by disrupting signal coherence.

Channel uncertainties in RIS-assisted covert communication arise from the following four main sources: (1) Shadowing: Random path loss variations due to obstacles; (2) Rayleigh Fading: Rapid amplitude/phase fluctuations in multipath environments; (3) RIS Phase Errors: Deviations in phase-shift adjustments from ideal values; (4) CSI Imperfection: Noisy estimates of PT-RIS–warden channel links. These uncertainties degrade the legitimate SNR by 3–5 dB and increase detection error variability, addressed here via worst-case performance analysis and robust phase-shift optimization.

Large-scale path loss $\left(m_{p,p}\right)$ is characterized by the equation

(2) $$\begin{array}{c} m_{p,p}=\beta_{0}d_{p,p}^{-\alpha_{1}}, \end{array}$$

where $\left(\beta_{0}\right)$ denotes the path-loss constant at the reference distance, $\left(\alpha_{1}\geq 2\right)$ represents the path loss exponent, and $\left(d_{p,p}\right)$ stands for the separation distance between PT and PR. In terms of small-scale fading, a Rayleigh fading model $\left(g_{p,p}∼\mathrm{CN}\left(0,\beta_{p,p}\right)\right)$ is adopted.

The RIS is able to capture signals sent by the PT. Each element of the RIS adjusts the phase shift of incoming signals via a controller. Then these signals are reflected toward the PR. Channel characteristics for the PT-to-RIS and RIS-to-PR paths can be formulated as

(3) $$\begin{array}{cc} h_{I} & =\beta_{0}d_{P,I}^{-\alpha_{2}/2}g_{I}, \end{array}$$

(4) $$\begin{array}{cc} h_{P,I} & =\beta_{0}d_{P,I}^{-\alpha_{2}/2}g_{P}, \end{array}$$

where $\left(h_{I}∼\mathrm{CN}\left(0,I_{M}\right)\right)$ and $\left(gB∼\mathrm{CN}(0,I_{M})\right)$ are Rayleigh fading channel vectors. The parameter $\left(\alpha_{2}\right)$ is the path loss exponent for RIS–ground communications, while (dP,I) and (dP,I) correspond to the distances between PT-RIS and RIS-PR, respectively. The PT transmits a Gaussian signal denoted by (x[i]) (where i∈1,2,…,L) with ${\left(E(|x\left[i\right]\right|}^{2})=1)$. The variable (L) signifies the block length, which is bounded by $\left(L\leq L_{\max}\right)$ (the maximum allowable block length). The received signal at PR can be expressed as

(5) $$\begin{array}{c} y_{P}\left[i\right]=\sqrt{P_{p}}\left(h_{p,p}+g_{P}^{H}\Theta h_{I}\right)x\left[i\right]+n_{P}\left[i\right],\;i\in 1,2,\ldots ,L, \end{array}$$

where $\Theta =\mathrm{diag}\{\psi_{1}e^{j\theta_{1}},\psi_{2}e^{j\theta_{2}},\ldots ,\psi_{M}e^{j\theta_{M}}\}$ denotes the RIS phase-shift matrix. In this matrix, $\left(\theta_{n}\right)$ denotes the phase shift and $\left(\psi_{n}\right)$ represents the amplitude of the n-th element (with $\left(\psi_{1}=\ldots =\psi_{M}=1\right)$ assumed to maximize the SNR). The term $\left(P_{p}\right)$ refers to the PT’s transmission power, and $\left(n_{P}\left[i\right]∼\mathrm{CN}\left(0,\sigma_{b}^{2}\right)\right)$ is the additive white Gaussian noise (AWGN) at PR with noise power $\left(\sigma_{b}^{2}\right)$.

Accounting for finite block length, the maximum channel coding rate from the PT to PR is expressed as

(6) $$\begin{array}{c} R=\log_{2}\left(1+\gamma_{b}\right)-\sqrt{\frac{V}{L}}Q^{-1}\left(\delta \right), \end{array}$$

where $\left(\gamma_{b}=\frac{P_{p}{\left|h_{p,p}+g_{P}^{H}\Theta h_{I}\right|}^{2}}{\sigma_{b}^{2}}\right)$ represents the SNR received at PR, (δ≥0) denotes the decoding error probability, $\left(V=\frac{\gamma_{b}\left(\gamma_{b}+2\right)}{{\left(\gamma_{b}+1\right)}^{2}}\right)$ stands for channel dispersion, and $\left(Q^{-1}\left(\cdot \right)\right)$ is the Q-function inverse. Effective throughput is defined by the equation

(7) $$\begin{array}{c} \eta =\rho_{1}LR\left(1-\delta \right), \end{array}$$

where $\rho_{1}$ and $\rho_{0}$ are the prior probabilities of transmission or silence, respectively, $\rho_{1}+\rho_{0}=1$. Throughput under covertness constraints is referred to as effective covert throughput (ECT).

## 4. Detection Performance for Willie

In this section, Willie covert communication detection mechanisms are examined using channel modeling, two-valued hypothesis checking, detection threshold optimization, and error probability analysis. In particular, PT-Willie channel and RIS-Willie channel modeling are used, and the optimal threshold to minimize detection errors using relativistic checking is obtained. The results show that UTPP has a lower detection error probability than ETPP. This presents a major challenge for covert communication and highlights the need to simultaneously optimize transmission parameters.

### 4.1. Signal Transmission to Willie

Willie aims to detect the PT’s transmission by analyzing the received signal power, employing an optimal detector to minimize errors. The channel between the PT and Willie is modeled similarly to the PT-PR link, incorporating both large-scale path loss and small-scale fading.

(8) $$\begin{array}{c} h_{p,w}=m_{p,w}g_{p,w}, \end{array}$$

where $m_{p,w}=\sqrt{\beta_{0}d_{p,w}^{-\alpha_{1}}}$ represents large-scale path loss (with ($d_{p,w}$) as the PT-Willie distance, typically larger than ($d_{p,p}$)), and ($g_{p,w}∼\mathrm{CN}\left(0,\beta_{p,w}\right)$) denotes Rayleigh fading. The RIS-Willie channel is defined by the vector ($h_{I,W}=\beta_{0}d_{I,W}^{-\alpha_{2}/2}g_{W}$), where $d_{I,W}$ denotes the RIS-Willie distance and $g_{W}∼\mathrm{CN}\left(0,I_{M}\right)$ represents the fading vector. When PT is transmitting, the received signal at Willie is

(9) $$\begin{array}{c} y_{W}\left[i\right]=\sqrt{P_{p}}\left(h_{p,w}+g_{W}^{H}\Theta h_{I}\right)x\left[i\right]+n_{W}\left[i\right], \end{array}$$

where ($n_{W}\left[i\right]∼\mathrm{CN}\left(0,\sigma_{w}^{2}\right)$) is additive white Gaussian noise (AWGN) at Willie.

### 4.2. Detection Threshold Derivation

Willie performs binary hypothesis testing to determine the PT’s state: ($H_{0}$): The PT is silent (only noise received). ($H_{1}$): The PT is transmitting (signal + noise received). The decision statistic ($T_{L}$) is defined as the average received power over (L) channel uses. Willie compares ($T_{L}$) to a threshold ($\Gamma_{L}$): if ($T_{L}>\Gamma_{L}$), ($H_{1}$) is declared (transmission detected); otherwise, ($H_{0}$) is assumed (no transmission).

Two types of errors occur as follows: False Alarm (FA): ($\mathrm{Pr}(D_{1}|H_{0})$), where ($D_{1}$) indicates a “transmit” decision under ($H_{0}$). Missed Detection (MD): ($\mathrm{Pr}(D_{0}|H_{1})$), where ($D_{0}$) indicates a “silent” decision under ($H_{1}$). The total detection error probability (DEP) is

(10) $$\begin{array}{c} P_{\mathrm{DEP}}=\rho_{0}\cdot \mathrm{Pr}\left(T_{L}>\Gamma_{L}|H_{0}\right)+\rho_{1}\cdot \mathrm{Pr}\left(T_{L}<\Gamma_{L}|H_{1}\right). \end{array}$$

The fixed threshold analysis here serves as a baseline; the optimal threshold derivation, accounting for dynamic channel conditions and prior probabilities, is presented in Section 4.3.

### 4.3. Optimal Threshold Derivation

To minimize ($P_{\mathrm{DEP}}$), Willie optimizes ($\Gamma_{L}$). Under ($H_{0}$), the received signal is pure noise, so ($T_{L}$) follows a chi-squared distribution ($\chi^{2}\left(2L,\sigma_{w}^{2}/L\right)$). Under ($H_{1}$), the signal includes both the PT’s transmission and noise, leading to ($T_{L}∼\chi^{2}\left(2L,\left(\kappa +\sigma_{w}^{2}\right)/L\right)$), where ($\kappa =P_{p}{\left|h_{p,w}+g_{W}^{H}\Theta h_{I}\right|}^{2}$).

Using likelihood ratio testing, the optimal threshold ($\Gamma_{L}^{*}$) is derived to balance FA and MD probabilities. It is shown that ($\Gamma_{L}^{*}$) depends on ($P_{p}$), prior probabilities ($\rho_{0}/\rho_{1}$), and channel parameters. For example, when ($\rho_{1}\neq \rho_{0}$) (UTPP), ($\Gamma_{L}^{*}$) shifts to favor the more probable hypothesis, reducing ($P_{\mathrm{DEP}}$) compared to ETPP.

With increasing $P_{p}$, the minimum $P_{\mathrm{DEP}}$ is reduced because stronger signals enhance the distinguishability between $H_{0}$ and $H_{1}$. UTPP outperforms ETPP in minimizing $P_{\mathrm{DEP}}$, thereby intensifying challenges to the PT’s covertness. Such findings underscore the necessity of jointly optimizing transmission parameters to counteract Willie’s detection capabilities.

## 5. Maximizing ECT in Conventional Scenarios

In this section, we examine strategies to maximize the ECT of RIS support systems in ETPP/UTPP mode. We construct a general optimization framework for phase-shift parameters, extend the model to Poisson distribution packet-generation scenarios, and analyze the power and block length trade-offs. According to the analysis of the results, by dynamically adjusting the transmission probability distribution, UTPP is possible to achieve a flexible balance between the communication rate and covert requirements. It is found that with ETPP, it is possible to achieve better performance.

### 5.1. ECT Maximization

When Willie employs the optimal detection threshold ($\Gamma^{*}$) under equal transmit prior probabilities ($\rho_{0}=\rho_{1}=0.5$), the minimum DEP is denoted as ($\xi_{1}^{*}$). The PT’s objective is to disrupt Willie’s detection accuracy by elevating ($\xi_{1}^{*}$) to satisfy the covertness constraint:

(11) $$\begin{array}{c} 2\xi_{1}^{*}\geq 1-2\varepsilon , \end{array}$$

where (ε) represents the covertness tolerance. Leveraging the total variation (TV) distance between probability distributions ($P_{1}$) (PT transmitting) and ($P_{0}$) (PT silent), the constraint translates to ($\mathrm{TV}(P_{1},P_{0})\leq 2\varepsilon$). Using Pinsker’s inequality, this is further relaxed to a relative entropy constraint ($D(P_{1}|P_{0})\leq 2\varepsilon$), where ($D(P_{1}|P_{0})$) quantifies the divergence between ($P_{1}$) and ($P_{0}$). The optimization framework for maximizing ECT under equal prior probabilities (ETPP) is formulated as

(12) $$\begin{array}{cc} \; & \max_{\begin{array}{c} P_{p},L,\Theta \end{array}}\eta \end{array}$$

(13) $$\begin{array}{cc} s.t.\; & \frac{\sqrt{L}P_{p}\vartheta }{4\sigma_{w}^{2}}\leq \varepsilon , \end{array}$$

(14) $$\begin{array}{cc} \; & 0<P_{p}\leq P_{\max}, \end{array}$$

(15) $$\begin{array}{cc} \; & 0<L\leq L_{\max}, \end{array}$$

where ($\eta =\rho_{1}LR\left(1-\delta \right)$) denotes the effective throughput, (*R*) is the coding rate, and (ϑ) aggregates channel fading and path loss parameters. The RIS phase-shift matrix (Θ) is optimized to maximize PR’s received SNR by aligning phases of direct and reflected signals, expressed as ($\theta_{n}=\arg\left(h_{p,p}\right)-\arg\left(g_{B,n}\right)-\arg\left(h_{I,n}\right)$).

The covertness constraint creates a trade-off between (*L*) (block length) and ($P_{p}$) (transmit power), requiring ($LP_{p}={\left(\frac{4\sigma_{w}^{2}\varepsilon }{\vartheta }\right)}^{2}$). ECT is maximized by using the maximum allowable block length ($L^{*}=L_{\max}$), with ($P_{p}$) adjusted to satisfy the covertness constraint: ($P_{p}^{*}=\min\left(\frac{4\sigma_{w}^{2}\varepsilon }{\sqrt{L_{\max}\vartheta }},P_{\max}\right)$).

For UTPP, the covertness constraint is redefined to account for asymmetric transmission probabilities $\rho_{1}$ and $\rho_{0}=1-\rho_{1}$. The constraint becomes $\left[\max\left(\rho_{0},\rho_{1}\right)\cdot D\left(P_{1}|P_{0}\right)\leq 2\varepsilon \min\left(\rho_{0},\rho_{1}\right)\right]$. This introduces flexibility in balancing transmission opportunities and covertness. The optimization problem under UTPP extends P1 by including $\rho_{1}$ as a variable:

(16) $$\begin{array}{cc} P2:\; & \max_{\begin{array}{c} P_{p},L,\rho_{1},\Theta \end{array}}\eta \end{array}$$

(17) $$\begin{array}{cc} s.t.\; & \frac{\max\left(\rho_{0},\rho_{1}\right)\sqrt{L}P_{p}\vartheta }{2\sigma_{w}^{2}}\leq 2\varepsilon \min\left(\rho_{0},\rho_{1}\right), \end{array}$$

(18) $$\begin{array}{cc} \; & 0<P_{p}\leq P_{\max}, \end{array}$$

(19) $$\begin{array}{cc} \; & 0<L\leq L_{\max}, \end{array}$$

(20) $$\begin{array}{cc} \; & \rho_{\min}\leq \rho_{1}\leq \rho_{\max}. \end{array}$$

The solution to P2 involves classifying cases based on ($\rho_{1}\leq \rho_{0}$) or ($\rho_{1}>\rho_{0}$).

Case 1 ($\rho_{1}\leq \rho_{0}$): The optimal ($\rho_{1}$) converges to 0.5 when ($\rho_{\min}\leq \frac{A}{1+A}\leq 0.5$) (where (*A*) is a channel-dependent constant), with ($P_{p}^{*}$) constrained by ($P_{\max}$). Case 2 ($\rho_{1}>\rho_{0}$): ($\rho_{1}$) is optimized to balance throughput and covertness, with ($P_{p}^{*}$) adjusted based on whether ($\rho_{1}$) is bounded by ($\rho_{\max}$) or channel conditions. Numerical results confirm that UTPP outperforms ETPP by enabling a more flexible trade-off between ECT and covertness, particularly when ($\rho_{1}$) is adjusted to exploit channel uncertainty at Willie.

The optimal block length is consistently ($L_{\max}$) in both ETPP and UTPP scenarios, as increasing channel use maximizes throughput, while ($P_{p}$) is constrained to maintain covertness. UTPP introduces an additional degree of freedom (prior probability adjustment), leading to higher ECT compared to ETPP under the same covertness constraints. RIS phase optimization plays a dual role, which involves enhancing PR’s SNR and introducing randomness at Willie, thereby reinforcing covertness.

### 5.2. Maximizing ECT in Packet-Generation Scenarios

In IoT networks, status update mechanisms are prevalent, where devices periodically transmit data packets upon sensing new information. The PT operates under a Poisson packet arrival process with rate ($\lambda_{a}$), meaning the probability of transmitting a packet in a time slot is ($\rho_{1}=1-e^{-\lambda_{a}T}$), and the silence probability is ($\rho_{0}=e^{-\lambda_{a}T}$). Here, (T=L/B) denotes the transmission delay, with (*B*) as the bandwidth and (*L*) as the block length. The objective is to maximize ECT by co-optimizing transmit power ($P_{p}$), block length (*L*), and RIS phase shifts (Θ). The optimization framework accounts for the coupling between ($\rho_{1}$) and (*L*) due to the Poisson arrival model, leading to the following problem:

(21) $$\begin{array}{cc} \; & \max_{\begin{array}{c} P_{p},L,\Theta \end{array}}\eta \end{array}$$

(22) $$\begin{array}{cc} s.t.\; & \frac{\max\left(\rho_{0},\rho_{1}\right)\sqrt{L}P_{p}\vartheta }{4\sigma_{w}^{2}}\leq \varepsilon \min\left(\rho_{0},\rho_{1}\right), \end{array}$$

(23) $$\begin{array}{cc} \; & 0<P_{p}\leq P_{\max}, \end{array}$$

(24) $$\begin{array}{cc} \; & 0<L\leq L_{\max}. \end{array}$$

For ETPP (($\rho_{0}=\rho_{1}=0.5)$), the Poisson arrival model imposes ($\lambda_{a}T=\ln2$); hence, ($L=B\ln2/\lambda_{a}$). Substituting into the covertness constraint, the optimal transmit power is derived as

(25) $$\begin{array}{c} P_{p}^{*}=\min\left(\frac{4\sigma_{w}^{2}\varepsilon }{\sqrt{L}\vartheta },P_{\max}\right). \end{array}$$

ECT is maximized by prioritizing the highest possible ($P_{p}$) under the covertness constraint, with (L) fixed by the ETPP condition. UTPP introduces two scenarios based on the relationship between ($\rho_{0}$) and ($\rho_{1}$):

($\rho_{0}\geq \rho_{1}$) (Low Packet Arrival Rate)

Here, ($\lambda_{a}T\leq \ln2$), limiting ($L\leq B\ln2/\lambda_{a}$). The covertness constraint becomes

(26) $$\begin{array}{c} \frac{\left(e^{\lambda_{a}L/B}-1\right)\sqrt{L}P_{p}\vartheta }{4\sigma_{w}^{2}}\leq \varepsilon . \end{array}$$

The optimal block length ($L^{*}$) and power ($P_{p}^{*}$) satisfy

(27) $$\begin{array}{cc} L^{*} & =\min\left(L_{\max},\frac{B\ln2}{\lambda_{a}}\right), \end{array}$$

(28) $$\begin{array}{cc} P_{p}^{*} & =\min\left(\frac{4\sigma_{w}^{2}\varepsilon \left(e^{\lambda_{a}L^{/}B}-1\right)}{\sqrt{L^{\vartheta }}},P_{\max}\right). \end{array}$$

($\rho_{0}<\rho_{1}$) (High Packet Arrival Rate)

For ($\lambda_{a}T>\ln2$), (*L*) exceeds ($B\ln2/\lambda_{a}$). The covertness constraint is adjusted to

(29) $$\begin{array}{c} \frac{\left(e^{\lambda_{a}L/B}-1\right)\sqrt{L}P_{p}\vartheta }{4\sigma_{w}^{2}}\leq \varepsilon . \end{array}$$

The optimal solution involves balancing (*L*) and ($P_{p}$) to maximize (η), with ($L^{*}$) determined by solving the first-order condition of the ECT function. Low-Latency Advantage: UTPP allows for shorter block lengths compared to ETPP, better satisfying IoT latency requirements. Saturation Behavior: ECT saturates with ($L_{\max}$) due to covertness constraints, while ($P_{p}^{*}$) is bounded by ($P_{\max}$). Poisson Rate Impact: Higher ($\lambda_{a}$) reduces the saturation block length ($L^{*}$), as frequent packet arrivals increase detection risk at Willie. This framework demonstrates that UTPP outperforms ETPP in dynamic packet-generation scenarios by flexibly adjusting transmission probabilities and block lengths to balance throughput and covertness.

## 6. Simulation Results

In this section, we demonstrate the effectiveness of the support-based covert communication strategy through simulation results. Specifically, we focus on key performance indicators including DEP, relative entropy, and ECT and systematically evaluate how core parameters—transmission power, block length, UTPP, and Poisson packet arrival rate—influence system performance. The results show that introducing RIS significantly improves ECT performance compared to non-RIS baselines, while UTPP outperforms ETPP by flexibly balancing transmission rate and covertness. Furthermore, this analysis reveals the following three critical findings: (1) an inverse relationship between the optimal transmission power and block length; (2) ECT saturation under maximum power and block length constraints; and (3) a decrease in the optimal block length for high Poisson arrival rates. These practical observations provide valuable insights for parameter optimization in RIS-assisted covert communication systems.

**Figure 2:**
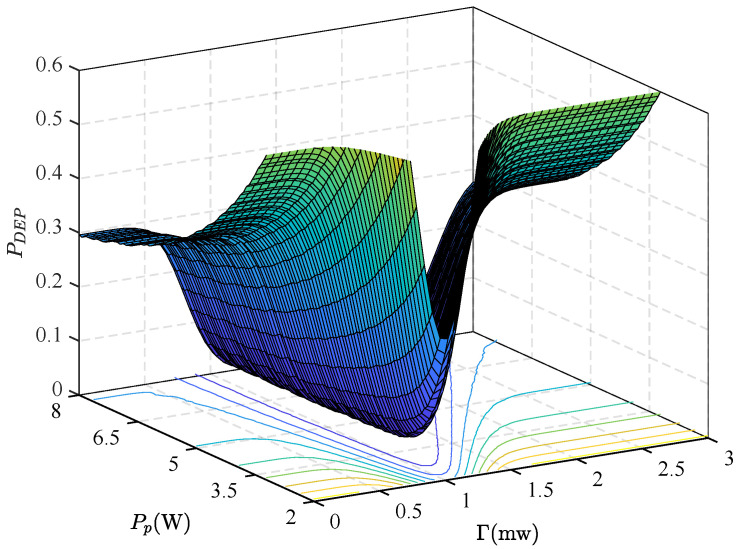
DEP versus $P_{P}$ achieved by different Γ.

Figure 2 illustrates the relationship between the detection threshold, false detection probability, and transmission power for the primary channel’s transmitter and detector. As shown, false detection probability decreases gradually with increasing PT, which means that a higher transmit power enhances signal strength, facilitating hidden target detection and reducing false detection. Concurrently, the false detection rate exhibits a non-monotonic trend with rising detection thresholds: it first decreases, then increases. A too low threshold triggers alarms from background noise alone, causing higher false detection probability, while an excessively high threshold weakens the detector’s ability to identify strong targets, also increasing false detection. Thus, selecting an appropriate detection threshold can optimize this balance, minimizing the false detection probability.

**Figure 3:**
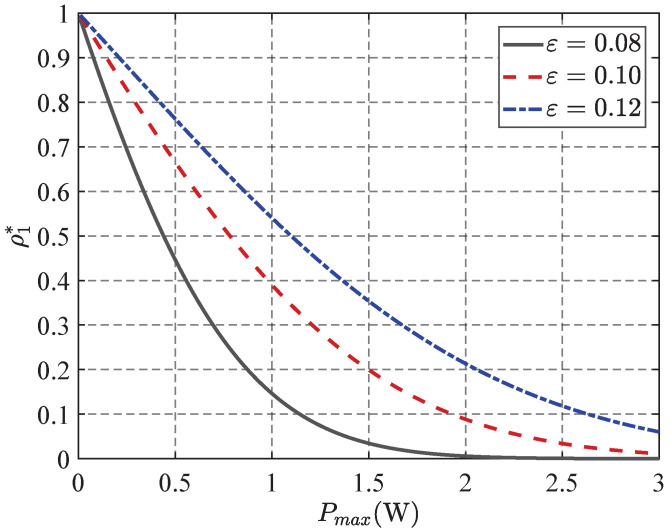
Transmission probability $\rho_{1}^{*}$ versus $P_{\max}$ achieved by different ϵ.

In Figure 3, the simulation results show how the transmission probability varies with maximum transmit power (PT) for different covert communication protocols. As can be seen, the transmission probability initially decreases with increasing transmit power, then stabilizes at higher transmit power levels. This trend is due to the fact that a higher PT increases the risk of detection, necessitating a reduction in the transmission probability to maintain covertness. However, when the PT exceeds a certain threshold, the detection risk peaks within the given covert constraint, and the transmission probability remains constant.

In addition, the three curves in the figure correspond to different covert constraint levels. It can be seen that stricter constraints (indicating a more conservative transmission strategy) lead to lower overall transmission probability and the need to further restrict transmission frequency in the high-power range.

**Figure 4:**
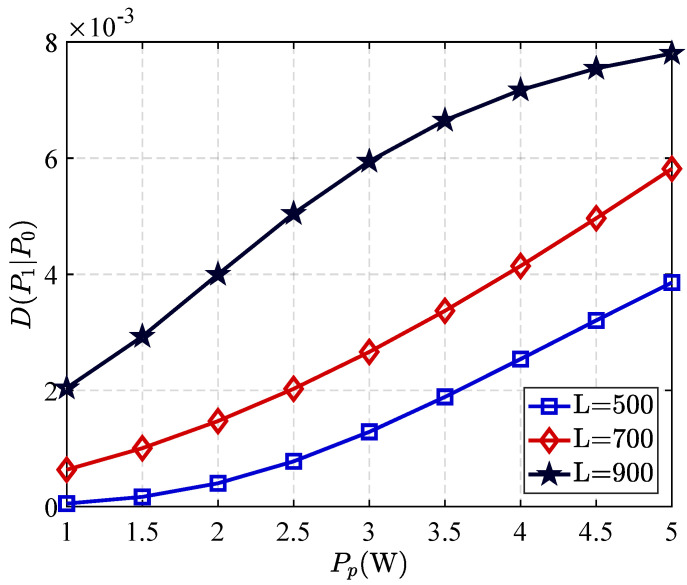
Relative entropy versus $P_{P}$ with different *L*.

In Figure 4, we present curves for three different block lengths, illustrating the relationship between the primary transmitter’s (PT) transmit power and relative entropy. It is readily apparent that the relative entropy at Willie increases monotonically with increasing PT transmit power. This occurs because higher transmit power enhances the adversary’s (Willie’s) signal observation capability.

Furthermore, the relative entropy exhibits a strong dependence on block length. This indicates that, at any fixed transmit power level, relative entropy increases with increasing block length. This phenomenon can be explained by the fact that longer observation intervals enhance Willie’s statistical recognition and detection performance.

**Figure 5:**
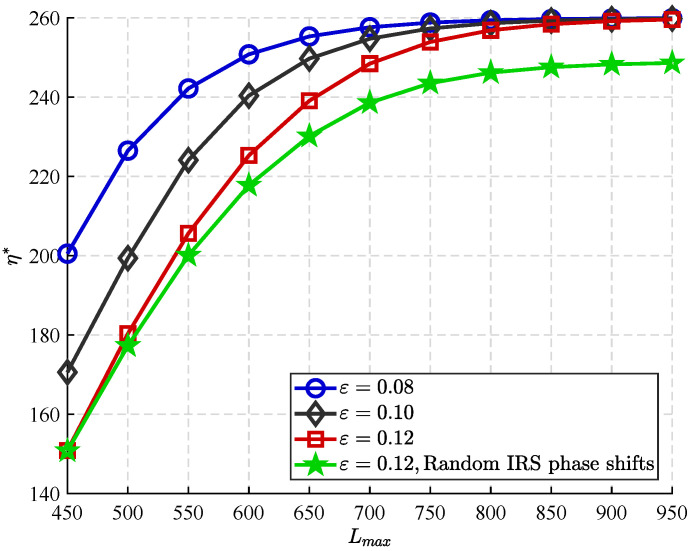
$\eta^{*}$ versus $L_{max}$ achieved by different ϵ.

In Figure 5, we present simulation results showing the correlation between the maximum block length constraint and the ECT, as well as the impact of varying covert constraint stringency. It is clear that the ECT increases monotonically with the maximum block length, as longer blocks enhance the statistical distinguishability of the primary transmitter (PT)’s transmitted signal. This improvement in distinguishability increases the probability of Willie detection while also increasing the ECT under the covert constraint. In addition, Figure 5 shows a curve labeled “Random RIS Phase Shifts” to serve as a baseline for validating the proposed optimization strategy. This baseline corresponds to scenarios where RIS phase shifts are randomly generated, simulating unoptimized passive beamforming. By comparing with the proposed scheme, the figure shows the performance gains from active RIS phase alignment, which jointly maximizes the legitimate receiver’s SNR while degrading signal quality at the warden.

Furthermore, the figure shows a strong correlation between the ECT and the level of covert constraint: as the constraint is tightened, the ECT systematically decreases for all block lengths. This decrease can be explained by the fact that stricter covert requirements limit the PT’s ability to efficiently transmit data without detection, thereby offsetting the throughput gains from increased block length. Thus, the curves in the figure are ordered by constraint stringency: within the range of block lengths examined, the strictest constraints correspond to the curves with the lowest ECT, while the loosest constraints correspond to the curves with the highest ECT.

**Figure 6:**
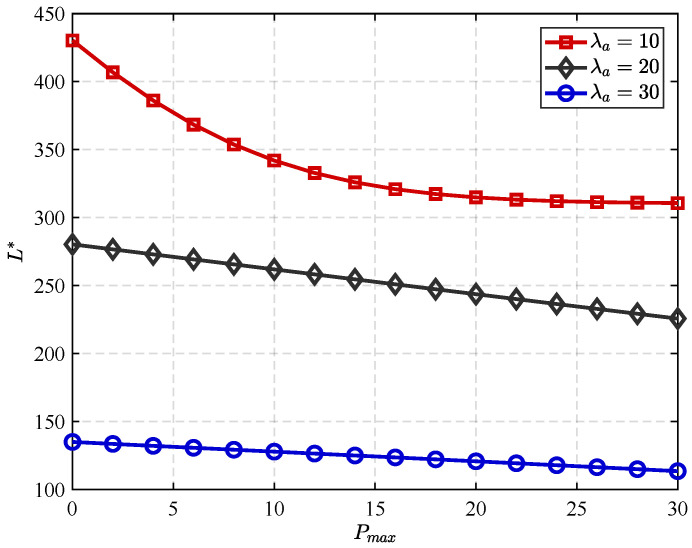
$L^{max}$ versus $P_{max}$ achieved by different $\lambda_{a}$.

In Figure 6, we show the optimal transmit power and the corresponding optimal block length for different Poisson arrival rates. It is easy to see that the optimal block length decreases monotonically with increasing transmit power. This trend occurs because higher transmit power improves information transmission efficiency, thereby reducing transmission time.

Furthermore, it is not difficult to see that the optimal block length is closely related to the Poisson arrival rate: at a fixed transmit power, the optimal block length systematically decreases with increasing arrival rate. This is because as the arrival rate increases, the probability of successfully initiating a transmission within a given interval also increases, thereby reducing the reliance on long blocks for reliable communication.

**Figure 7:**
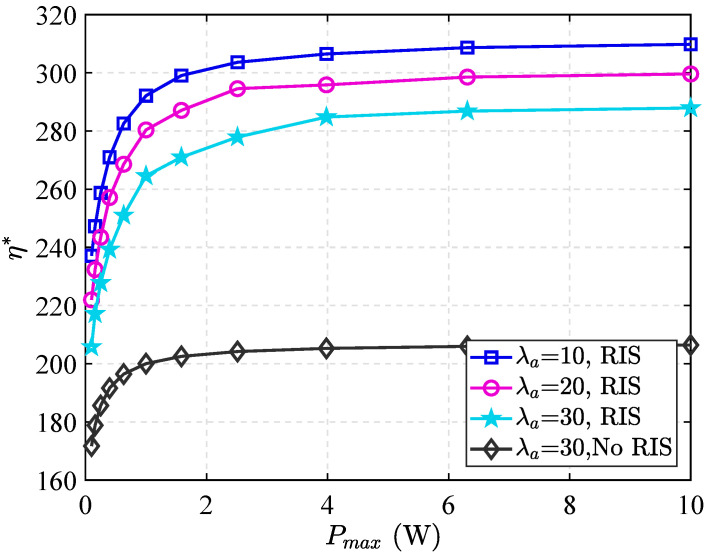
$\eta^{*}$ versus $P_{\max}$ achieved by different $\lambda_{a}$.

In Figure 7, we experimentally demonstrate the relationship between the ECT of the PT and its maximum transmit power. It can be seen that the ECT increases monotonically with transmit power until it reaches a saturation threshold. Beyond this threshold, further increases in power do not improve performance. This saturation phenomenon occurs because, while a stronger signal initially improves throughput, fundamental system-specific constraints ultimately limit further gains.

Furthermore, it is readily apparent that the transmission probability is positively correlated with transmit power within the evaluated PT power range. This trend suggests that a higher transmission probability increases the adversary’s probability of detection. Notably, when the RIS is not deployed, the ECT is lowest at all power levels, demonstrating the critical role of the RIS in improving the system’s ECT.

## 7. Conclusions

We propose a comprehensive framework for robust covert communication in CRNs by strategically deploying RISs. To address the core challenge of statistically hiding SU transmissions from enemy guards while strictly minimizing harmful interference to PUs, we propose a novel approach to optimize passive beamforming parameters by jointly optimizing SU transmit power, communication block length, and RISs. The RIS is a legitimate SU. It aims to achieve two goals, namely improving signal reception quality at the receiver while degrading it at the guards and alleviating the square root law trade-off between covertness and throughput.

In summary, this paper proposes an RIS-based framework to enhance the security and spectral efficiency of next-generation dynamic spectrum sharing systems, laying the foundation for high-performance covert communications in CRNs. In the future, we will explore the design of wireless communication security system frameworks for scenarios with multiple monitors, imperfect channel state information, and practical hardware limitations.

## Data Availability

Data are contained within the article.
